# Sensory Disturbances, but Not Motor Disturbances, Induced by Sensorimotor Conflicts Are Increased in the Presence of Acute Pain

**DOI:** 10.3389/fnint.2017.00014

**Published:** 2017-07-21

**Authors:** Clémentine Brun, Martin Gagné, Candida S. McCabe, Catherine Mercier

**Affiliations:** ^1^Center for Interdisciplinary Research in Rehabilitation and Social Integration Québec, QC, Canada; ^2^Department of Rehabilitation, Laval University Québec, QC, Canada; ^3^Royal National Hospital for Rheumatic Diseases Bath, United Kingdom; ^4^Department of Nursing and Midwifery, University of the West of England Bristol, United Kingdom

**Keywords:** body image, body schema, acute pain, virtual reality, sensorimotor integration

## Abstract

Incongruence between our motor intention and the sensory feedback of the action (sensorimotor conflict) induces abnormalities in sensory perception in various chronic pain populations, and to a lesser extent in pain-free individuals. The aim of this study was to simultaneously investigate sensory and motor disturbances evoked by sensorimotor conflicts, as well as to assess how they are influenced by the presence of acute pain. It was hypothesized that both sensory and motor disturbances would be increased in presence of pain, which would suggest that pain makes body representations less robust. Thirty healthy participants realized cyclic asymmetric movements of flexion-extension with both upper limbs in a robotized system combined to a 2D virtual environment. The virtual environment provided a visual feedback (VF) about movements that was either congruent or incongruent, while the robotized system precisely measured motor performance (characterized by bilateral amplitude asymmetry and medio-lateral drift). Changes in sensory perception were assessed with a questionnaire after each trial. The effect of pain (induced with capsaicin) was compared to three control conditions (no somatosensory stimulation, tactile distraction and proprioceptive masking). Results showed that while both sensory and motor disturbances were induced by sensorimotor conflicts, only sensory disturbances were enhanced during pain condition comparatively to the three control conditions. This increase did not statistically differ across VF conditions (congruent or incongruent). Interestingly however, the types of sensations evoked by the conflict in the presence of pain (changes in intensity of pain or discomfort, changes in temperature or impression of a missing limb) were different than those evoked by the conflict alone (loss of control, peculiarity and the perception of having an extra limb). Finally, results showed no relationship between the amount of motor and sensory disturbances evoked in a given individual. Contrary to what was hypothesized, acute pain does not appear to make people more sensitive to the conflict itself, but rather impacts on the type and amount of sensory disturbances that they experienced in response to that conflict. Moreover, the results suggest that some sensorimotor integration processes remain intact in presence of acute pain, allowing us to maintain adaptive motor behavior.

## Introduction

To maintain accurate movements that are adapted to the outside world, the sensory feedback arising from our actions is systematically compared to our motor intentions (Blakemore et al., 2000; Frith et al., 2000). While this function is critical to detect unexpected perturbations, correct for inadequate planning and support motor learning, Harris (1999) has proposed that a discordance between motor intention and sensory feedback (creating a sensorimotor conflict) may result in the sensation of pain acting as an “error signal”. The most obvious example that has been proposed by Harris to illustrate this theory is the case of phantom limb pain, in which the intention to move the phantom limb cannot result in appropriate sensory feedback from the missing body part. However, sensorimotor conflicts can also arise when the limb is still present. For instance, in complex regional pain syndrome a conflict can arise between the intended movement (e.g., completely open the hand) and the limited movement that can actually be performed (McCabe and Blake, 2008). Interestingly, canceling out the discordance between motor intention and visual feedback (VF) by spatially superposing a mirror image of the non-painful limb on the painful limb can alleviate pain in various chronic pain populations (Ramachandran and Rogers-Ramachandran, 1996; McCabe et al., 2003; Mercier and Sirigu, 2009; McCabe, 2011). In contrast, creating an experimental sensorimotor conflict with a mirror can transiently exacerbate painful sensations and other sensory disturbances (as feelings of peculiarity, loss of control, perceived extra limb or loss of limb, changes in weight or temperature) (McCabe et al., 2007; Daenen et al., 2010, 2012). Changes in motor performance have also been observed during exposure to the mirror feedback, but reports have focused more on the sensory consequences of those changes in performance rather than recording a specific trajectory change (McCabe et al., 2005, 2007). For example, participants with and without chronic pain describe a loss of awareness and control of limbs, and the researchers observe altered limb trajectory and poor bilateral alignment of the limbs (McCabe et al., 2005, 2007).

In healthy volunteers, sensorimotor conflicts produced experimentally generate the same type of sensory disturbances (Daenen et al., 2010; Foell et al., 2013; Roussel et al., 2015), but to a lesser extent to what is observed in chronic pain populations (McCabe et al., 2007; Daenen et al., 2010). These conflicts have even been reported to sometimes induce painful sensations (McCabe et al., 2005), although this remains controversial (Foell et al., 2013; Don et al., 2017). Less robust body representations in the presence of pain could contribute to explain this difference in the intensity of the response to sensorimotor conflicts between individuals with chronic pain and healthy individuals, as well as altered sensory perception and motor performance that are often observed in chronic pain populations (Lotze and Moseley, 2007; Nijs et al., 2012).

Indeed various chronic pain states are associated with alterations in sensory perception and body representations, such as an overestimation of the size of the painful limb (Lewis et al., 2007; Peltz et al., 2011; Nishigami et al., 2015), an altered sense of position (Gelecek et al., 2006; Moseley, 2008; Lewis et al., 2010) and movement (Roosink et al., 2015). These alterations in body representations have sometimes been reported to be associated with the severity of motor disturbances observed in chronic pain populations (Bank et al., 2013; Hamacher et al., 2016). In healthy volunteers, the induction of experimental acute pain can also transiently alter body representations, as shown by shifts in the subjective body midline toward the painful side (Bouffard et al., 2013), overestimation of the size of the painful limb (Gandevia and Phegan, 1999) and altered sense of position (Eva-Maj et al., 2013) Finally, patients with chronic pain have altered somatosensory (Flor et al., 1997; Di Pietro et al., 2013) and motor (Lotze et al., 2001; Maihöfner et al., 2007) cortical representations. A recent study has shown that the presence of acute pain enhances the corticospinal excitability changes induced by subsequent transient deafferentation of the hand (Mavromatis et al., 2016). Together, these results support the view that pain might make the body representations more plastic, both at the cortical and perceptual level.

The general objective of this study was to assess sensory and motor disturbances induced by sensorimotor conflicts, and to test whether these disturbances are influenced by the presence of experimental pain. We hypothesized that both sensory and motor disturbances would be increased in presence of pain, which would support the idea that pain makes body representations less robust. To test this hypothesis, participants realized cyclic asymmetric movements of flexion-extension with both upper limbs in a robotized system combined with a 2D virtual environment. The virtual environment allowed the provision of VF about movements that were either congruent or incongruent, while the robotized system allowed precise measurement of motor performance (in addition to subjective perception of the participant), before and during exposure to different types of VF. The effect of experimental pain was compared to three control conditions (no somatosensory stimulation, tactile distraction and proprioceptive masking) to ensure that the effect of pain was not due to a simple distraction or to an impact of pain on the integration of proprioceptive information. Indeed, integration of proprioceptive information has been reported to be altered in the presence of pain. (Lee et al., 2010; Sheeran et al., 2012; Eva-Maj et al., 2013).

A secondary objective was to determine whether the amount of sensory disturbances induced by sensorimotor conflicts was associated with the extent of motor disturbances.

## Materials and methods

### Participants and ethics statement

Thirty healthy caucasian participants (26 right-handed as determined in the Edinburgh Inventory Test (Oldfield, 1971)—15 females—mean ± SD age: 27.7 ± 5.9 years) were recruited from Laval University. None of them had a history of visual, nervous system or musculoskeletal disease that could affect task performance. All participants provided their written informed consent prior to admission to the study. The experiment was performed in accordance with the tenets of the Declaration of Helsinki and the study protocol was approved by the local ethical review board (Institut de réadaptation en déficience physique de Québec, Canada, n°2015-461).

### Study design

**Figure 1:**
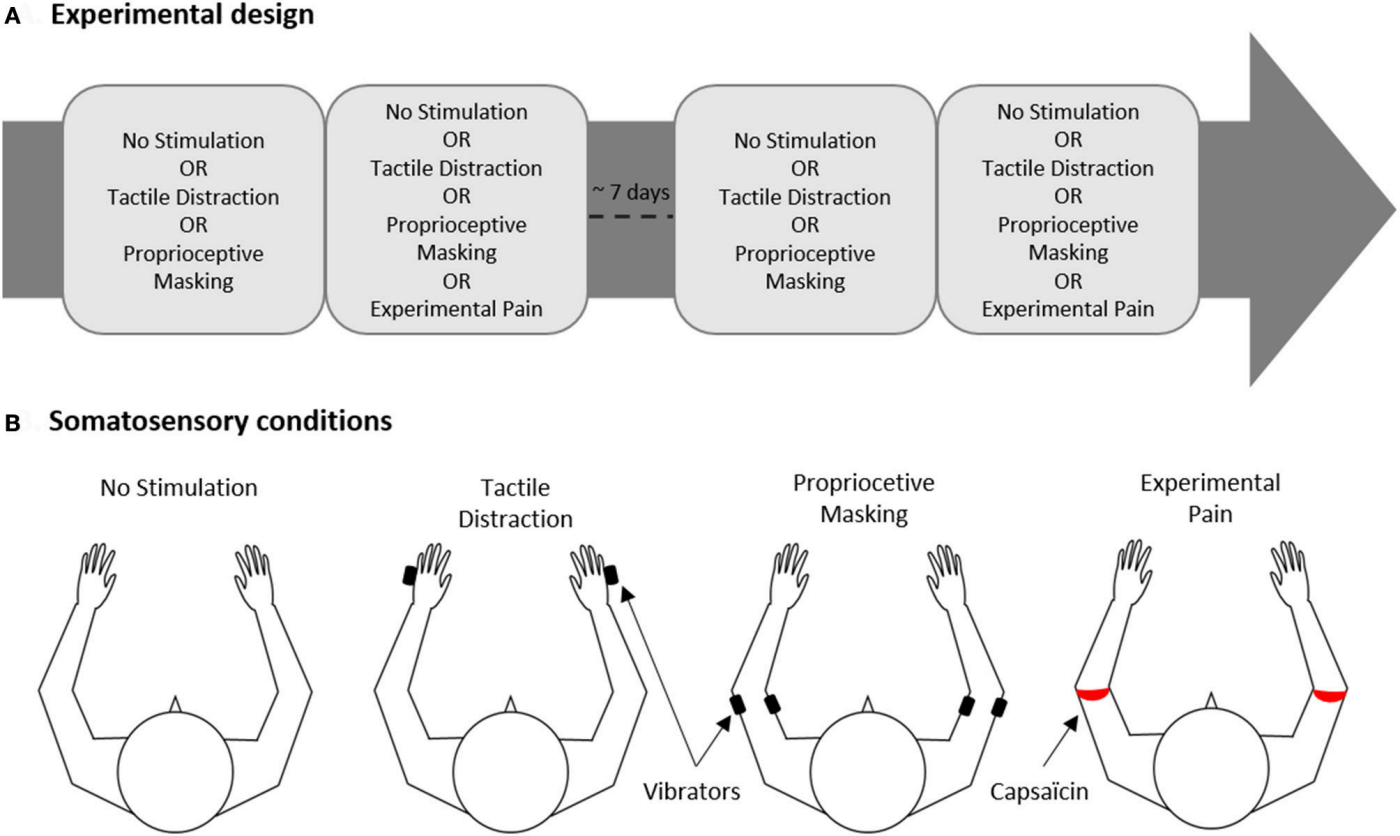
Experimental design. **(A)** The experiment was carried out on two experimental sessions separated by ~7 days for each participant. Each session comprised two blocks of trials, one block corresponded to one of the four somatosensory conditions. **(B)** Somatosensory conditions. Black and red rectangles indicate the site of application of vibrators or capsaicin cream, respectively.

The experiment was conducted on two experimental sessions separated by 6.9 ± 2.7 days (Figure 1A). In total, each participant was exposed to 16 experimental conditions (described in details in Section Experimental Conditions) presented in a factorial within-subject design: [*Somatosensory conditions* (“No Stimulation” or “Tactile Distraction” or “Proprioceptive Masking” or “Experimental Pain”)] × [*Visual conditions* (“Congruent VF” or “No VF” or “Flipped VF” or “Mirror VF”)].

**Figure 2:**
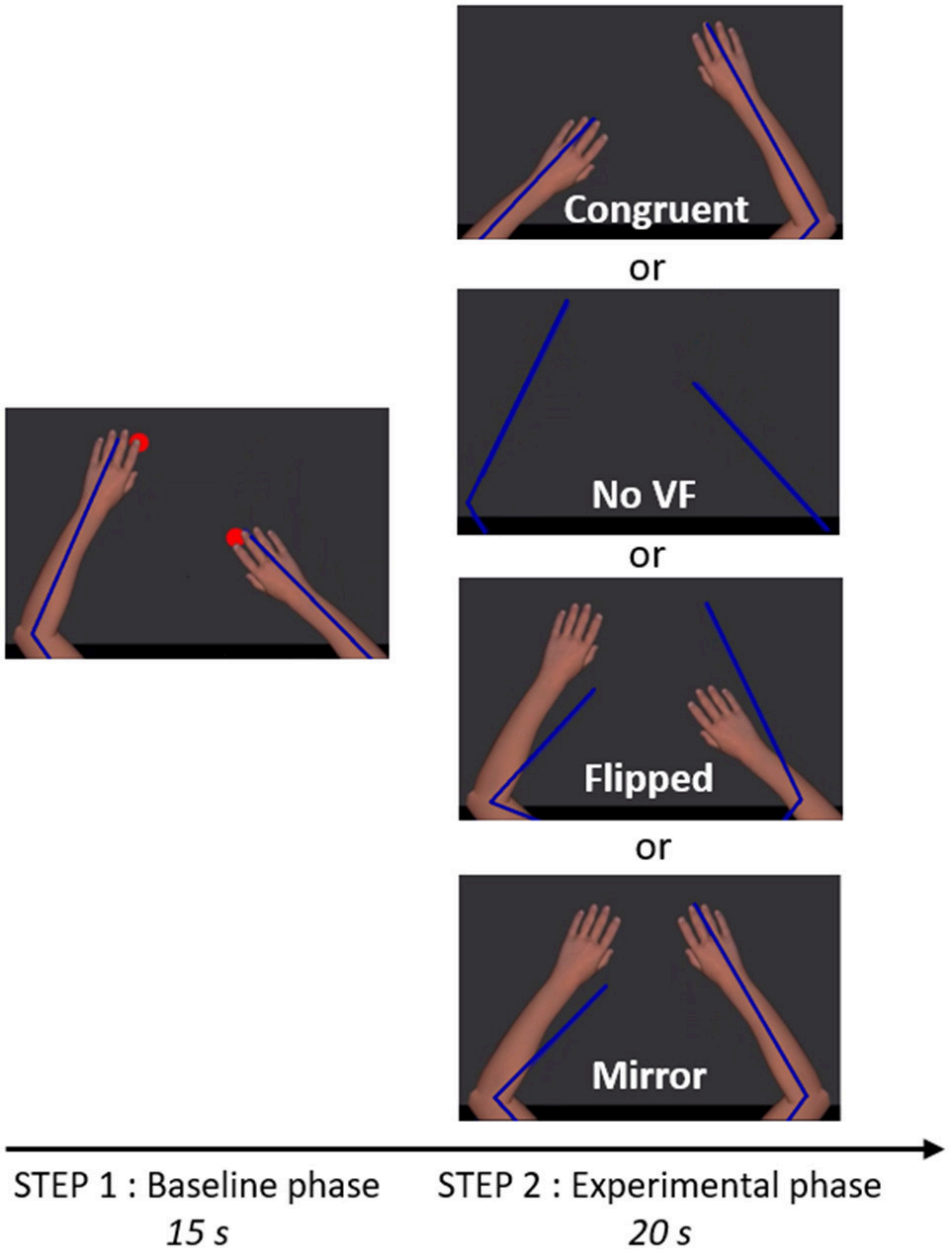
Trial timeline and visual conditions. The participant saw exclusively the virtual upper limbs (Step 1 and 2) as well as the red targets (Step 1). Blue lines depict the real position of the upper limbs. The size and the center of rotation of virtual upper limbs were adjusted to correspond to the real upper limbs of the participant. In Step 1, two red targets were alternating in anti-phase at 1.25 Hz and participants were instructed to reach successively toward the targets, in order to create a bilateral anti-phase movement. In Step 2, the red targets were disappearing and one of the four visual conditions depicted was presented, providing either congruent or incongruent visual feedback (VF) about the limb movement.

In each of the two sessions, participants realized two blocks of trials, each block corresponding to one of the four somatosensory conditions (No Stimulation, Tactile Distraction, Proprioceptive Masking or Experimental Pain, Figure 1B). Each block included 8 trials, i.e., two trials of each of the 4 visual conditions (Congruent VF, No VF, Flipped VF or Mirror VF, see Figure 2) presented in a pseudo-random order. Note that given that the effect of experimental pain (induced with capsaicin) does not vanish immediately after the removal of the capsaicin cream, the Experimental Pain condition was systematically the last block in the session. However, the order to the four somatosensory conditions was counterbalanced in such a way that the average rank of all somatosensory conditions was similar. Each participant performed a total of 32 experimental trials (4 Visual conditions X 4 Somatosensory conditions X 2 trials) over the two sessions (i.e., 16 trials by session).

### Instrumentation and experimental task

**Figure 3:**
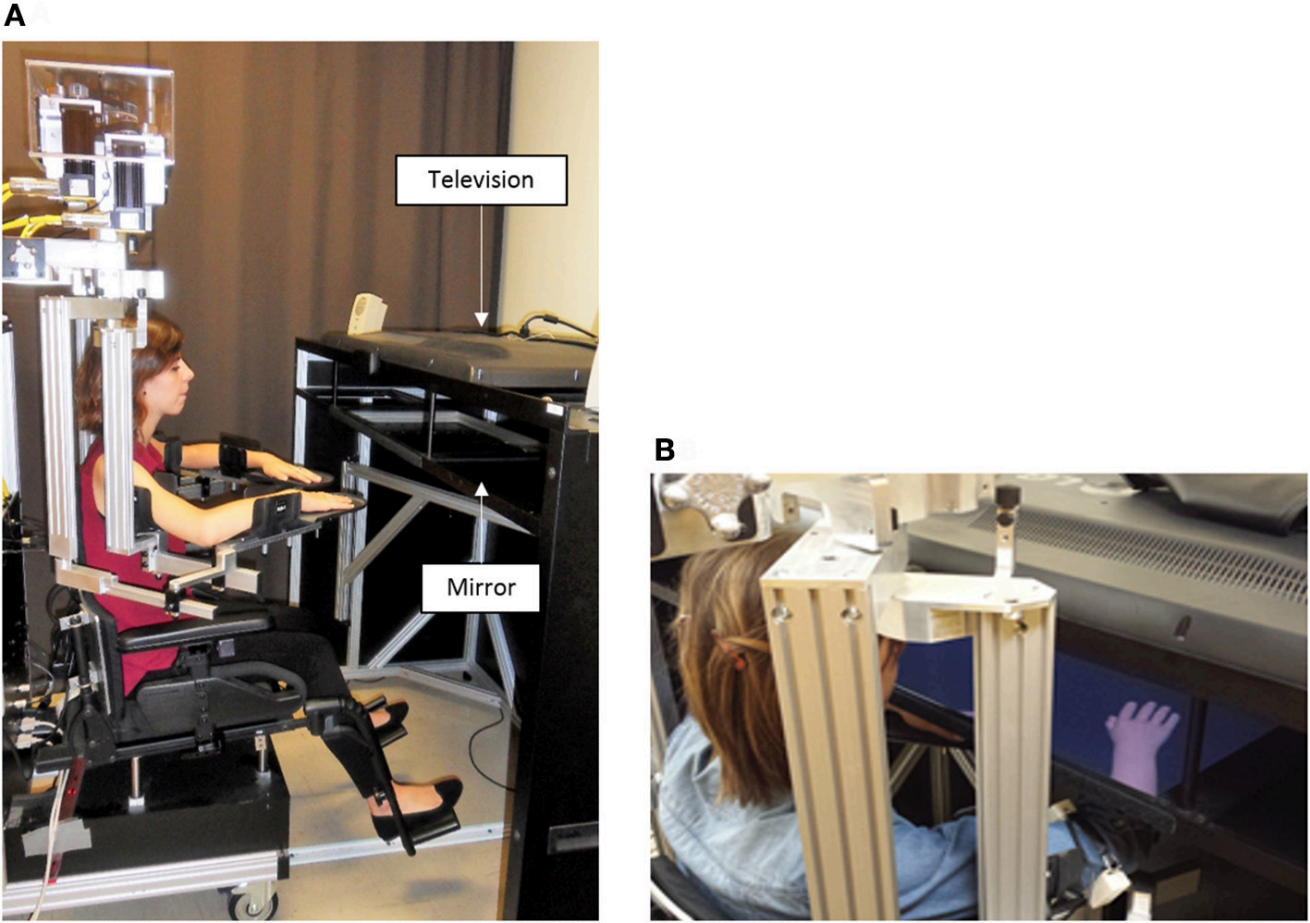
Experimental set up. Exoskeleton robot **(A)** and 2D virtual environment **(B)** are the 2 elements of the KINARM. The exoskeleton is fitted to the anthropometric characteristics of the participant. The virtual environment consists in the projection of virtual upper limbs on a semi-transparent mirror (47″) thanks to a television. Upper limbs rest on the exoskeleton under the semi-transparent mirror and are obstructed from the participant' view.

The experiment was conducted using the KINARM (BKIN Technologies, Kingston ON, Canada; see Figure 3A), a robotized bilateral exoskeleton that allows combined movements of the shoulder (horizontal abduction-adduction) and elbow (flexion-extension) joints in order to move upper limbs (ULs) in the horizontal plane. A 2D virtual environment (47″) created the illusion of two virtual ULs replacing participant's ULs (with appropriate vision of depth), that were always obstructed from view (Dexterit-E software version 3.4.2; Figure 3B). These virtual ULs that were driven by participant' ULs in real-time provided the possibility to manipulate the VF given to the participant in a much more flexible manner than the mirror box set-up that is typically used in this type of experiments: we had the possibility to program virtual ULs to move synchronously or asynchronously with the real movement of the participant, or to disappear, giving us an ideal scenario to create varied sensorimotor conflicts while recording the impact of these conflicts on the movement of each UL. Joint angular positions for both the shoulder and elbow were obtained from KINARM motor encoders and sampled at 1 kHz, and the position of the index was computed in real-time. Data processing was made with Matlab (MathWorks, R2011b).

Each session began with two trials of familiarization with the motor task (35 s each), in which the virtual ULs reproduced faithfully the movement of the participants' ULs (corresponding to Step 1 described below). Figure 2 illustrates the task that was then used throughout the experiment and comprised two steps. Before each trial, participants had to position their ULs on two green targets (2 cm of diameter) corresponding to an angular position of 85° for the elbows and 40° for the shoulders.

In Step 1 (baseline phase, 15 s), the virtual ULs were always congruent with the position of the real ULs. Two red targets (2 cm diameter) were appearing and alternating in anti-phase at 1.25 Hz. Location of the red targets was 10 cm anterior or posterior to the position of the green targets. Participants were instructed to reach successively toward red targets, in order to create a bilateral anti-phase movement (i.e., when one UL was in its peak of flexion, the other was in its peak of extension, with an endpoint movement amplitude of 20 cm in the antero-posterior axis for each UL). Participants were instructed to execute a fluid movement, without stopping on the red targets. To help participants to follow the rhythm, a metronome beat the time every 800 ms. Red targets were only present during this step, in order to help the participant to achieve the expected movement amplitude, but the metronome beat was maintained until the end of Step 2 to help keeping the rhythm. After the baseline phase (Step 1) and just before the experimental phase (Step 2), the red targets and the virtual ULs were disappearing for 0.8 s, while the metronome was still beating.

In Step 2 (experimental phase, 20 s), one of the four visual conditions (Congruent VF, No VF, Flipped VF, or Mirror VF) was presented. Except in the No VF condition, in which the screen remained completely black, participants were seeing their virtual ULs (although the position/movement was not necessarily congruent with their movements). Participants were instructed to continue to do the same movement and to always look at both virtual ULs, even if the visual condition was troubling (see Supplementary Material for a video of an experimental trial).

At the end of each trial, participants had to respond to questions about their sensations and perceptions in their ULs during the experimental phase.

### Experimental conditions

#### Visual conditions (present only in step 2)

Four visual conditions were studied (Figure 2), one control condition (Congruent VF) and three sensorimotor conflict conditions (No VF, Flipped VF and Mirror VF):

1. *Congruent VF*: the virtual UL reproduced faithfully the movements of the participant;
2. *No VF*: participants were watching a black screen (with eyes open);
3. *Flipped VF*: the left virtual UL reproduced the movement of the right UL and the right virtual arm reproduced the movement of the left UL;
4. *Mirror VF*: both virtual ULs reproduced the movement of the non-dominant UL.

#### Somatosensory conditions (present through both step 1 and step 2)

Four somatosensory conditions were studied (Figure 1B):

1. *No stimulation*: no somatosensory stimulation applied to the ULs;
2. *Tactile Distraction*: a 40-Hz vibration was applied on each hand (5th metacarpal; irrelevant to the task performed) to control for attentional effects related to the application of somatosensory stimuli to ULs;
3. *Proprioceptive Masking* was achieved by co-vibrating the biceps and triceps of both ULs at 40 Hz. Vibration preferentially activates muscle spindle endings (Roll and Vedel, 1982). When the vibration is applied to a single muscle, illusory movements or a reflexive contraction can be elicited (Calvin-Figuière et al., 1999). However, when the vibration is applied equally to agonist-antagonist muscles these effects are canceled out and the co-vibration method degrades proprioceptive responsiveness (Bock et al., 2007). Although the preferential frequency used is 80 Hz (Bock et al., 2007), 40 Hz is sufficient to degrade proprioception (Cordo et al., 1995; Chancel et al., 2016) without inducing discomfort (to maintain a clear distinction with the Experimental Pain condition). When vibrators were installed, biceps and triceps were first stimulated separately to evoke an illusory movement of extension and flexion, and then we ensured that co-vibration canceled out these illusory movements. All participants except one reported illusory movements when the biceps or triceps were stimulated separately, and this illusion was always canceled out during co-vibration;
4. *Experimental Pain* was induced with a single topical application of 1% capsaicin cream. A thin layer (~1 mm) of cream forming a 1 cm ring was applied around the upper arm, just proximal to the elbow, on both ULs. This location was selected because elbow joints were the most directly involved in the motor task performed by the subject, and that location was visible on the virtual ULs. Moreover, the fact that capsaicin was applied all around the arm creates a penetrating and irradiating burning sensation, which aimed to reproduce neuropathic pain. When the capsaicin cream was applied, participants were required to verbally rate their pain intensity using a numeric pain rating scale (NPRS) ranging from 0 (no pain) to 10 (the worst pain imaginable). Experimental block began when the pain reached an intensity of 5/10 for both ULs, or when the pain reached a plateau (average of 18 ± 4 min). The average of pain intensity reported at the beginning of the experimental block was 5.4 ± 1.6 for the left arm, 5.3 ± 1.6 for the right arm, and did not differ between both ULs (*p* = 0.96).

### Measures and data analysis

Each outcome was expressed as a change from the baseline phase in order to cancel out any direct effect that the somatosensory condition could have had on the movement or the perception of the limb.

#### Sensory disturbances

After each trial, participants had to verbally answer to nine yes-no questions: “*In the last trial, when the red targets were not present, did you feel any change or the appearance of…?”* (i.e., dichotomic choice without intensity rating, but with the possibility to add comments). Questions were targeting perceptions of pain, discomfort, losing a limb, temperature change, weight change, having an extra limb, losing control, peculiarity or any other sensations. Participants had to report any changes from the baseline phase, meaning for example that in the Experimental Pain condition, participants were instructed to answer no to the question about pain if the pain level was similar between the baseline period and the experimental condition. This questionnaire is based on previous studies assessing the impact of sensorimotor conflict on sensory disturbances in healthy volunteers (McCabe et al., 2005; Foell et al., 2013) and in chronic pain populations (McCabe et al., 2007) using open questions. The descriptors obtained through open questions in these studies were used to produce yes-no question for the present study, allowing quantification of the changes induced in the sensations across conditions. However, the last question (to report any other changes) allowed participants to report changes that were not covered by the questionnaire. A total score for the nine items was computed, corresponding to the mean percentage of sensory disturbances. For one experimental condition, a score of 0% indicated that the participant experienced no sensory disturbances for the nine items on the two trials. A score of 100% indicated that the participant experienced sensory disturbances on every item in both trials.

#### Motor performance

Two main outcomes were used to assess motor performance, both based on the position of the endpoint (index finger):

1. *Amplitude asymmetry* between both upper limbs: y-coordinates for both indexes were encoded for each peak of flexion and extension. For each movement half-cycle, the amplitude on the y-axis was extracted for each UL, and then the absolute difference between both ULs was calculated (see **Figure 6** for an example).
2. *Medio-lateral drift*: for each movement half-cycle, the x-coordinate of the maximal deviant point was extracted. The difference between the highest (i.e., most lateral point) and the lowest (i.e., most medial point) values was then calculated to obtain the range of the medio-lateral drift. Values of both ULs were pooled because they did not differ (*p* = 0.19) (see **Figure 6** for an example).

The motor performance for both outcomes in the Baseline phase are presented in Supplementary Figure 1. Both motor outcomes were expressed as a change from the baseline phase in order to cancel out effects of the somatosensory condition, as we were not interested in the effect of vibration or pain on motor control *per se*, but rather on alteration in motor performance induced by the conflict. Such normalization was needed as there was a small, but significant, effect of somatosensory condition on the amplitude asymmetry (*p* < 0.01), less asymmetry being observed in Tactile Distraction condition compared to the three others (*p* < 0.05). No effect of somatosensory condition was observed for medio-lateral drift (*p* = 0.20). Change from baseline was calculated by subtracting the performance during the last 10 s of the baseline phase from the performance during experimental phase. A positive value indicates a degradation of motor performance (i.e., more interlimb amplitude asymmetry or more medio-lateral drift) and a negative value an improvement.

### Statistics

Sensory disturbances and the two motor outcomes were analyzed using 4 × 4 repeated-measures analyses of variance (rmANOVA). *Post-hoc* tests were performed using Tuckey's correction for multiple comparisons. Statistical significance was set at *p* < 0.05. *P*-values were Huynh–Feldt corrected for sphericity when necessary. Mean ± standard deviation are reported in the results. Statistical analysis was performed with R software (version 3.1.2).

To answer the secondary objective of the study—to determine whether the sensory disturbances induced by sensorimotor conflicts were associated with motor disturbances—participants were arbitrarily split into two equal groups (*n* = 15/group), that were named the Minimal and the High disturbances group (see **Figure 7A**). The three sensorimotor conflict conditions were pooled together to classify participants according to their sensitivity to conflicts during No Stimulation and Experimental Pain conditions. Then, the effect of Group on motor outcomes was tested with *t*-test.

## Results

### Sensory disturbances

**Figure 4:**
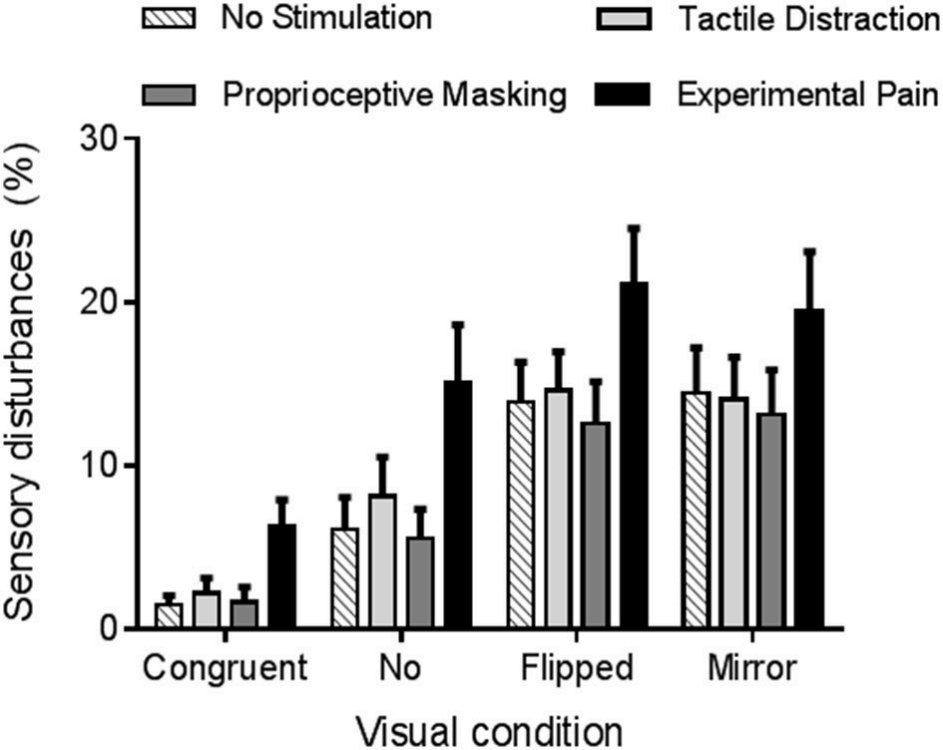
Average amount of sensory disturbances reported in each experimental condition. Error bars represent the standard error of the mean.

The rmANOVA revealed a strong main effect of vision (*p* < 0.0001, η_p_ = 0.42). As it can be seen on Figure 4, participants reported more sensory disturbances in conditions of sensorimotor conflicts (Flipped *VF* = 15 ± 14%, Mirror *VF* = 15 ± 16%, No *VF* = 9 ± 13%) than in Congruent VF (3 ± 5%, *p* < 0.05). Flipped and Mirror VF did not differ from each other (*p* = 0.99), but both induced more sensory disturbances than No VF condition (*p* < 0.05). Furthermore, a main effect of somatosensory condition was observed (*p* < 0.001, η_p_ = 0.24). Experimental Pain (15 ± 16%) induced more sensory disturbances than Proprioceptive Masking (8 ± 11%, *p* < 0.001), Tactile Distraction (10 ± 11%, *p* < 0.001) and No Stimulation (9 ± 11%, *p* < 0.001) conditions. Proprioceptive Masking, Tactile Distraction and No Stimulation conditions did not differ from each other (*p* > 0.75). Finally, no significant interaction was observed between somatosensory and visual conditions (*p* = 0.60).

**Figure 5:**
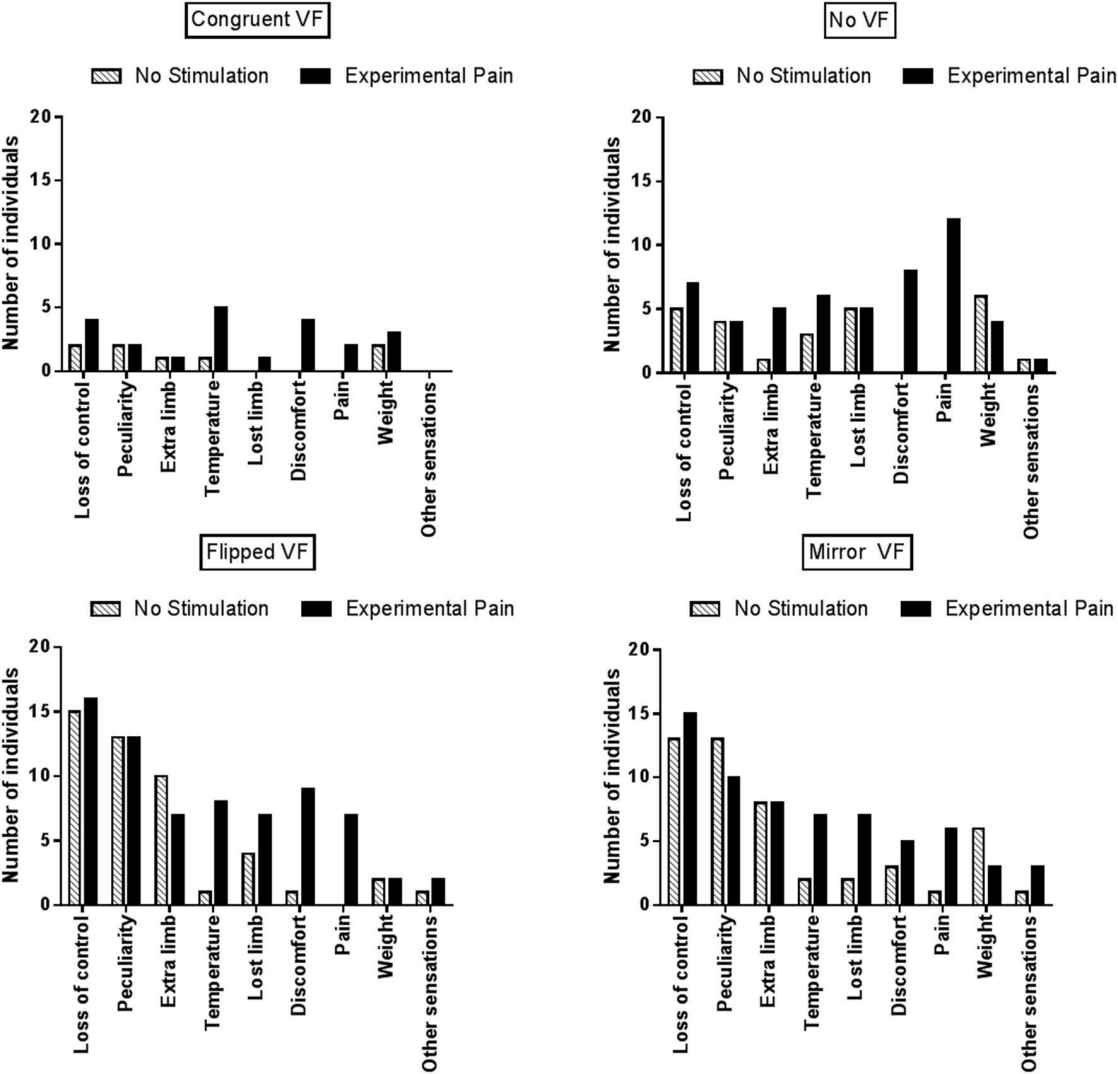
Number of individuals who reported at least one disturbance for a given item, reported as a function of the experimental condition.

As Proprioceptive Masking and Tactile distraction conditions did not differ from the No Stimulation condition, further comparisons focused solely on the differences between Experimental Pain and No Stimulation conditions. Figure 5 displays the number of individuals who reported each specific type of disturbance in each visual condition. Note that no statistical analyses were undertaken due to the large variability across items and the fact that the proportion of participants reporting a given item was often low: these results should therefore be considered as exploratory. During sensorimotor conflicts (No VF, Flipped VF, and Mirror VF), participants reported mainly a sensation of loss of control, peculiarity and the perception of having an extra limb. However, the occurrence of these three items was not influenced by the presence of Experimental Pain. It was rather the disturbances related to changes in intensity of pain or discomfort, changes in temperature (hotter or colder, depending on the participant) or the impression of a missing limb that appeared to differ between Experimental Pain and No Stimulation condition. Such effects were observed in the three conditions of sensorimotor conflict. Importantly very few participants reported disturbances related to changes in intensity of pain in the Congruent VF condition performed in the presence of Experimental Pain, which shows that the participants understood well that they were expected to report only pain increases, and not pain sensations *per se* (which were obviously present in all Experimental Pain trials). The other disturbances experienced by participants during sensorimotor conflicts were nausea, dizziness and numbness in the hand. Finally, when Experimental Pain was applied five participants reported the perception of an extra limb like “having a phantom hand” in the No VF condition.

### Motor performance

**Figure 6:**
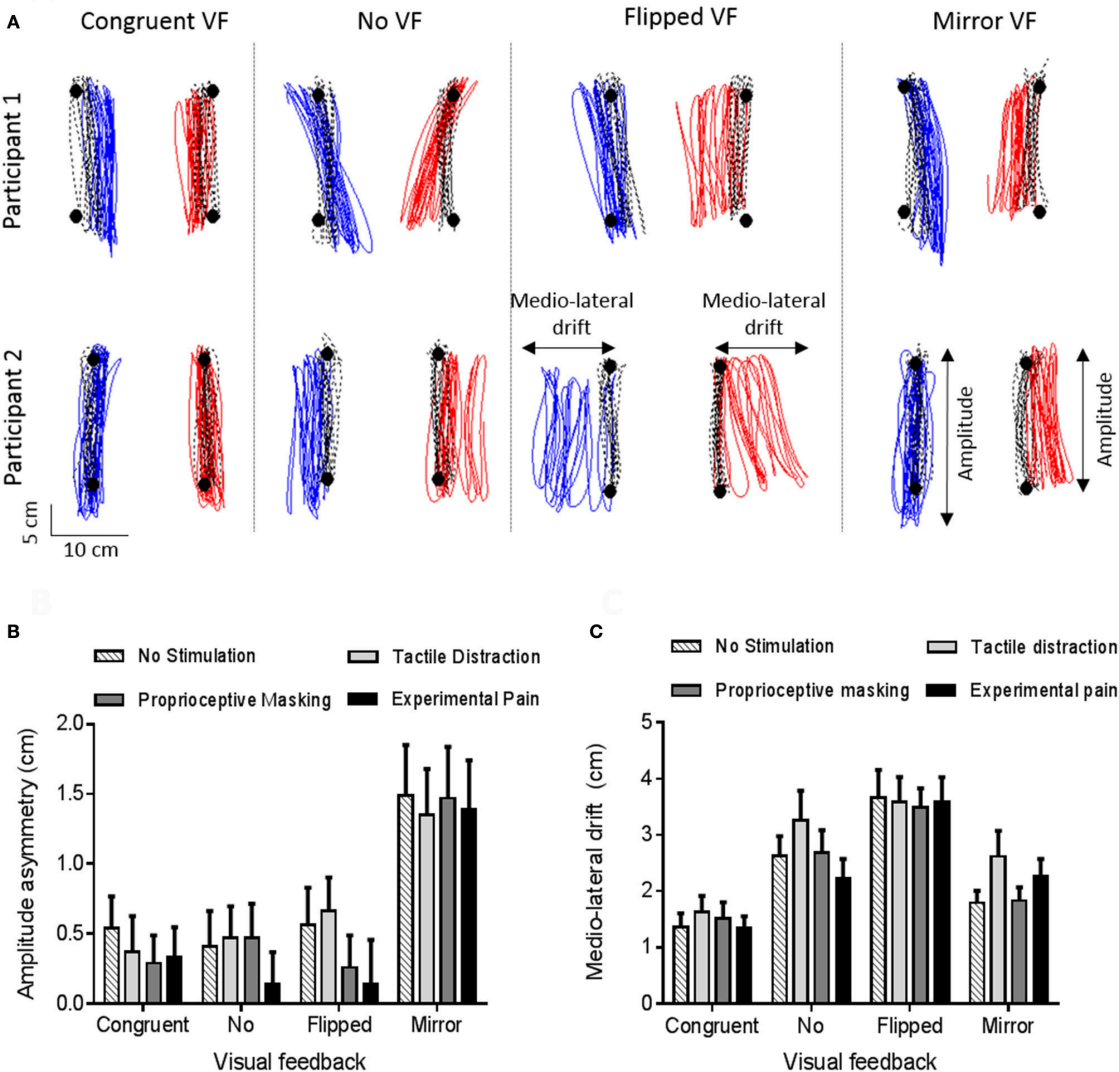
Motor disturbances. **(A)** Individual data for two representative participants, in absence of somatosensory stimulation. Black circles and black dashed lines represent, respectively, targets and trajectory of ULs during the baseline phase (Step 1). Blue and red lines represent, respectively, the trajectory of the left and right ULs during the experimental phase (Congruent VF, No VF, Flipped VF or Mirror VF—Step 2). **(B,C)** Amplitude asymmetry between left and right ULs **(B)** and medio-lateral drift **(C)**. A positive value indicates a degradation of motor performance relative to baseline. Error bars represent the standard error of the mean.

Figure 6A provides an example of motor disturbances induced by sensorimotor conflicts in the absence of somatosensory stimulation for two representative participants. Visual inspection of the data shows that motor disturbances are observed both in the antero-posterior and in the medio-lateral axis, and illustrates how the two motor outcome variables (Amplitude asymmetry and Medio-lateral drift) capture these disturbances. Moreover, we can see that motor disturbances differ according to the visual condition, and that some variability is present across participants. Finally, motor disturbances in the Congruent VF comparatively to the Baseline phase are observed and are explained by the fact that the red targets were disappearing during the Experimental phase (in order to avoid visual cues about errors in the conflict conditions).

#### Amplitude asymmetry

A significant main effect of vision (*p* < 0.0001, η_p_ = 0.29) was observed. As shown on Figure 6B, the asymmetry was larger in the Mirror VF condition (1.4 ± 1.9) than in the Congruent VF (0.4 ± 1.2, *p* < 0.0001), No VF (0.4 ± 1.3, *p* < 0.0001) and Flipped VF (0.4 ± 1.5, *p* < 0.0001) conditions. In Mirror VF, the dominant UL (for which incongruent VF was provided) did smaller movements than the non-dominant UL (for which congruent VF was provided). Congruent VF, No VF and Flipped VF conditions were not statistically different (*p* > 0.99). The somatosensory condition had no significant effect on Amplitude asymmetry (*p* = 0.54) and no significant interaction was observed between the somatosensory and the visual conditions (*p* = 0.93).

#### Medio-lateral drift

The ANOVA revealed a main effect of vision (*p* < 0.0001, η_p_ = 0.36). As shown on Figure 6C, participants drifted more in Flipped VF (3.6 ± 2.4 cm) than in the three other conditions (*p* < 0.05). Moreover, Mirror VF (2.1 ± 1.7 cm) and No VF (2.7 ± 2.2 cm) did not differ statistically from each other (*p* = 0.26), but only No VF differed statistically from Congruent VF (1.5 ± 1.4 cm, *p* < 0.05). However, there were no significant main effect of somatosensory conditions (*p* = 0.20) and no significant interaction between visual and somatosensory conditions (*p* = 0.31).

### Perception and motor performance

**Figure 7:**
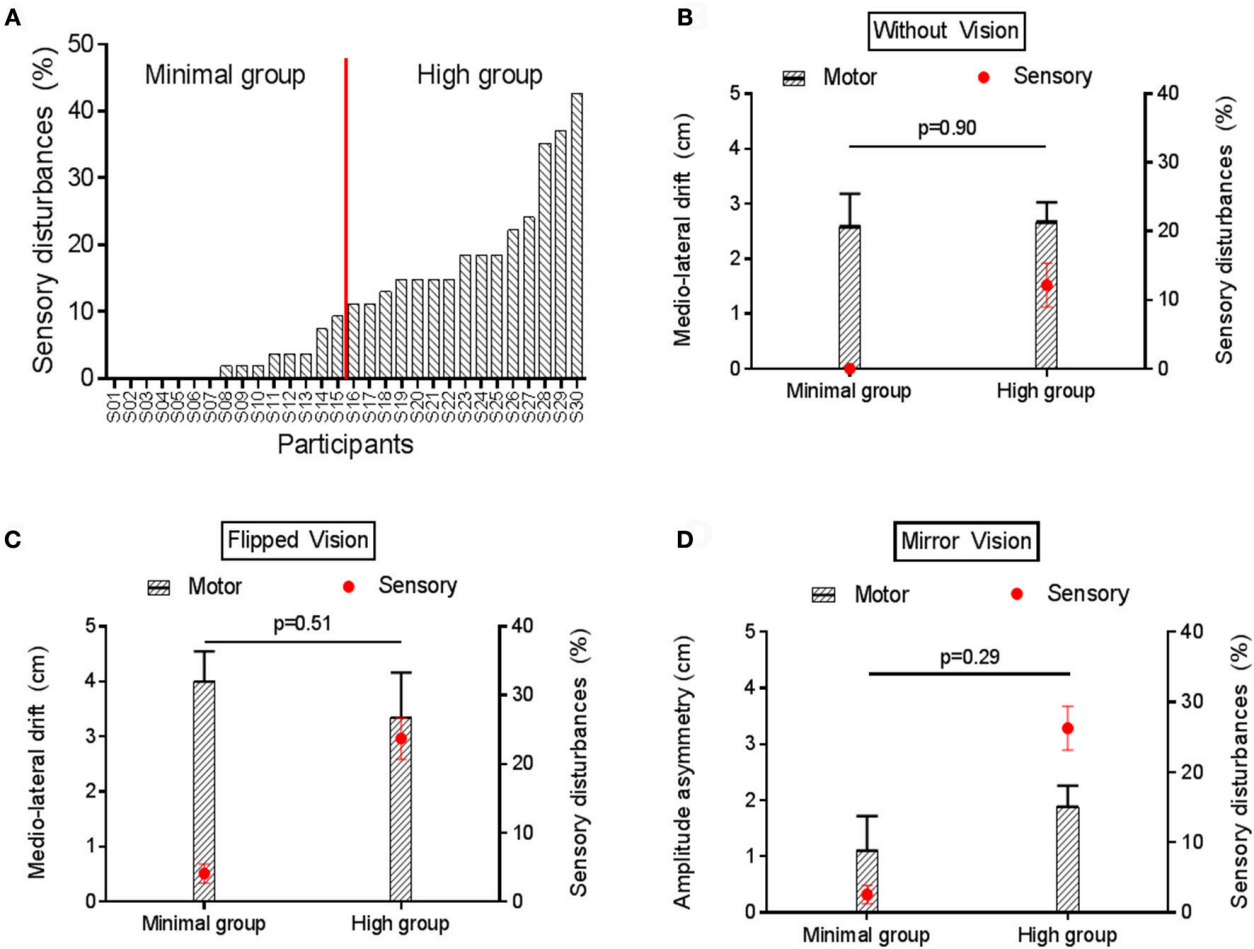
Motor and sensory disturbances induced by sensorimotor conflict in the No Stimulation condition. **(A)** represents the amount of sensory disturbances across participants for the three sensorimotor conflict conditions during No Stimulation condition. **(B,C)** compares the average medio-lateral drift (black bars) and amount of sensory disturbances (red circles) between groups with Minimal vs. High sensory disturbances, in the No VF and Flipped VF conditions, respectively. **(D)** compares the average amplitude asymmetry (black bars) and amount of sensory disturbances (red circles) between groups with Minimal vs. High sensory disturbances in the Mirror VF condition. Error bars represent the standard error of the mean. *P*-values are reported only for motor disturbances (as groups were formed based on amount of sensory disturbances).

Figure 7A shows the variability in the amount of sensory disturbances experienced across participants in condition of sensorimotor conflicts during No Stimulation somatosensory condition, ranging from 0% (no disturbances at all in the three sensorimotor conflict conditions) to 42.6%. Based on this average score of sensory disturbances, participants were arbitrarily split in to two equal groups to explore factors related to the sensitivity to sensorimotor conflicts as assessed by sensory disturbances. No difference was observed between the Minimal and the High disturbances group in terms of gender and age (*p* = 0.96). To explore whether groups also differed on the amount of motor disturbances, they were compared on the motor outcome that was the most sensitive to each type of conflict, i.e., the medio-lateral drift for No VF (Figure 7B) and Flipped VF (Figure 7C) conditions and amplitude asymmetry for the Mirror VF (Figure 7D). No significant difference was observed on motor performance between the Minimal and the High disturbances groups for any of the sensorimotor conflict in the No stimulation condition (Figure 7). The same result was observed for the Experimental Pain condition, (all *p* > 0.32; Supplementary Figure 2). Importantly, the intensity of pain reported by both groups following the application of capsaicin was similar (*p* = 0.84).

## Discussion

While previous studies on sensorimotor conflicts have focused only on evoked sensory disturbances in pain-free individuals and chronic pain patients, the two main original contributions of the present study were to investigate simultaneously sensory and motor disturbances evoked by such conflict, as well as to assess how they are influenced by the presence of acute pain. Results of the present study show that looking at virtual ULs which provide VF about movement that is incongruent with our actual movement induces sensory and motor disturbances in healthy participants. Contrary to what we hypothesized—that both motor and sensory disturbances would increase in the presence of pain—only sensory disturbances were enhanced during the experimental pain condition comparatively to the three control conditions (no stimulation, tactile distraction or proprioceptive masking). Moreover, this increase did not depend on the VF condition (congruent or incongruent). Finally, results show that motor and sensory disturbances induced by sensorimotor conflicts are not related with each other.

Sensory disturbances reported by the participants, involving mainly perceptions of loss of control, of peculiarity or of having an extra limb, are consistent with previous studies in which sensorimotor conflicts were induced with a mirror (McCabe et al., 2005; Daenen et al., 2010; Foell et al., 2013; Roussel et al., 2015). Sensory disturbances were also reported during the Congruent VF, but significantly less than in condition of sensorimotor conflicts. This could be explained by the fact that although virtual ULs were realistic in shape and adjusted to the arm's length of each participant, the match with the real arms was never perfect, thus creating a minor sensorimotor conflict. Moreover, our results showed that even in the absence of VF (i.e., when virtual ULs, present during Step 1, were suddenly disappearing during Step 2) participants reported more sensory disturbances than in the Congruent VF condition, but to a lesser extent than Flipped and Mirror VF. In line with this result, individuals with fibromyalgia report increased sensory disturbances when they close their eyes, including the perception of an extra limb. The induction of sensory disturbances by sensorimotor conflicts is a large and robust effect: 42% of the variance in sensory disturbances was explained by the visual condition in our study, and similar effects have been reproduced several times both in healthy participants (McCabe et al., 2005; Daenen et al., 2010; Foell et al., 2013; Roussel et al., 2015) and in chronic pain populations (McCabe et al., 2007; Daenen et al., 2010, 2012). It supports the idea that sensory disturbances might be acting as a warning signal when a discordance occurs between our motor intentions and the sensory feedback of the action. It has been suggested that a sensorimotor conflict can be sufficient to trigger painful sensations in healthy subjects (Harris, 1999; McCabe et al., 2005, 2009), but this remains controversial (Don et al., 2017). In our study, only one participant out of thirty reported painful sensations in the No Stimulation condition, supporting the idea that painful sensations can sometimes be elicited with sensorimotor conflicts in healthy individuals (McCabe et al., 2005), but that this is the exception rather than the rule.

The presence of acute pain, but not of other sensory manipulations, was found to enhance sensory disturbances in all visual conditions, including conditions of sensorimotor conflicts, consistent with the fact that chronic pain populations report more sensory disturbances than pain-free individuals (McCabe et al., 2007; Daenen et al., 2012). However, no statistically significant interaction between visual and somatosensory conditions was observed, which question whether this effect was specific to the situation of sensorimotor incongruence. An aspect that makes quantitative comparisons between conditions difficult in this type of study is the fact that although a large proportion of individuals report abnormal sensations in response to conflict, different types of disturbances are experienced and simply counting the number of sensations reported is certainly an imperfect approach. It is possible that some types of sensations (e.g., discomfort, pain, lost limb) reflect a higher degree of disturbance than others (e.g., change in weight or temperature). Interestingly, items that were the most sensitive to sensorimotor conflicts in the absence of pain (loss of control, feelings of peculiarity, perception to having an extra-limb) were not increased in the presence of experimental pain. New types of sensations (changes in intensity of pain or discomfort, changes in temperature or the impression of a missing limb) were rather appearing, mainly in the three conditions of conflict. This suggests that, contrary to our initial hypothesis, pain does not make individuals generally more sensitive to sensorimotor conflicts. Based on this hypothesis, we would have expected to see an increase in the frequency of reports of the type of sensations that were typically evoked by the conflicts in the absence of experimental pain. Our results rather suggest that while pain does not make people more sensitive to the conflict itself, it impacts on the type and amount of sensory disturbances that they experienced in response to that conflict. This idea is supported by a recent study comparing EEG cortical sources in healthy subjects under conditions of sensorimotor congruence or incongruence, while taking into account the amount of discomfort generated during sensorimotor incongruence (Nishigami et al., 2014). Interestingly, they reported that sensorimotor incongruence was associated with increased activation in the right posterior parietal cortex, irrespective of whether discomfort was experienced or not. However, individuals who were highly sensitive to discomfort exhibited more activation in two pain-related areas: anterior cingulate cortex and posterior cingulate cortex. In light of these results, we could hypothesize that in the presence of acute pain, the effect of the sensorimotor conflict on posterior parietal cortex activity would not be modified (i.e., no change in the sensitivity to conflict *per se*) but that the activity in pain-related areas would be increased, resulting in a different (and larger) set of sensory symptoms.

In contrast with our observations in acute pain, comparison between individuals with fibromyalgia and healthy controls suggests that chronic pain results in an increase in the frequency of reports of the disturbances that are typically evoked by sensorimotor conflict, in addition to sensory disturbances that appear to be more pain-specific (McCabe et al., 2007). This might indicate that chronic pain, but not acute pain, make individuals more sensitive to sensorimotor conflicts. This difference between acute and chronic pain could be explained by the fact that parietal dysfunctions has been reported in individual with chronic pain (Cohen et al., 2013; Kim et al., 2015), and that sensorimotor incongruence is associated with increased parietal activations (Nishigami et al., 2014).

Results on motor disturbances evoked by the sensorimotor conflicts also contradict the hypothesis that acute pain makes individuals generally more sensitive to sensorimotor conflicts. If it was the case, we would expect to see an impact of pain on both sensory and motor disturbances, while no effect of pain on motor disturbances was observed. This, and the observation that the amount of sensory disturbances perceived is not indicative of the amount of motor disturbances exhibited suggest that sensory and motor disturbances depend on different mechanisms. These results indirectly support the multiple body representations model, which dissociates the body schema governing the motor action, and the body image underlying the perceptual judgment. This theory was built according the Perception-Action model (Haffenden and Goodale, 1998) which suggests a dissociation between the “where”—*ventral pathway*—and the “what”—*dorsal pathway*. Although this theory of multiple body representations originally explained pathological cases like deafferentation or neglect syndrome (Paillard, 1999; De Vignemont, 2010), it had been shown that such dissociation also exists in healthy volunteers (Kammers et al., 2009). In light of that theory, our results would be interpreted as indicating that acute pain alters body image (perceptual judgment), but without impacting on body schema. This suggests that some sensorimotor integration processes remain intact in the presence of pain which allows us to maintain adaptive motor behavior, a view supported by two recent studies showing that acute pain does not interfere with sensorimotor integration as measured by short afferent inhibition paradigm (Burns et al., 2016; Mercier et al., 2016). However, it is possible that pain of a longer duration is needed to impact on body schema, given that movement disorders become more prevalent in complex regional pain syndrome as the disease progresses (Van Hilten, 2010).

Some limitations of the present study need to be highlighted. First, it is surprising that no effect of co-vibration was observed on motor performance, questioning whether proprioceptive masking was effectively achieved. Although the preferential frequency used for co-vibration is 80 Hz (Bock et al., 2007), we used a 40 Hz frequency to avoid inducing discomfort (to maintain that condition independent of the Experimental Pain condition). However, the lack of effect of bilateral co-vibration on motor performance does not necessarily indicate that proprioception was not degraded, as previous studies showing a degradation of bimanual coordination used co-vibration on only one UL, therefore creating an asymmetry on the proprioceptive feedback from both sides (Swinnen et al., 2003; Metral et al., 2014; Brun and Guerraz, 2015). Second, for the sensory perception questionnaire performing statistical analyses for each item was considered inappropriate in view of the large inter-subject variability, therefore these results should be interpreted cautiously. For future studies, using a scale that allows the assessment of the intensity of the disturbances (e.g., a Likert scale) rather than a binary answer (yes-no question) might provide more sensitivity. Another interesting approach would be to measure objectively the sensory disturbances induced by sensorimotor conflict, e.g., change in skin temperature (Moseley et al., 2013). Third, although all participants exhibited motor disturbances in presence of sensorimotor conflicts, the exact manner in which the movement disorganized was quite variable from one subject to another. Although we have been able to successfully identify motor outcomes that were sensitive to the visual condition, it is possible that these variables were not the most sensitive to the effect of pain. The use of a simpler motor task, for example a unilateral task (which is not possible to do with a mirror but could be achieved with virtual reality) or of a single-joint bilateral task, might allow to decrease inter-subject variability and therefore increase the sensitivity of the measure for future studies. Finally, tonic pain was used in order to mimic a neuropathic pain condition, as sensory disturbances are predominantly reported in populations with neuropathic pain. Using a phasic pain model, in which the occurrence of pain would be related to a specific movement of the participant, could have more impact on the motor disturbances.

In conclusion, acute pain does not appear to make people more sensitive to sensorimotor conflict itself, but rather impacts on the type and amount of sensory disturbances that they experience in response to that conflict. However, it needs to be kept in mind that the impact of acute pain on body representation might differ from that of chronic pain. Moreover, results showed no relationship between the amount of motor and sensory disturbances evoked in a given individual. This suggests that some sensorimotor integration processes remain intact in the presence of acute pain, allowing us to maintain adaptive motor behavior even though limb perception is altered.

## Author contributions

CM, CSM, and CB designed the study; CB and MG performed data collection; CB, MG, CSM, and CM analyzed and interpreted the data; CB and CM drafted the paper, CSM and MG commented on the paper and approved the final version.

### Conflict of interest statement

The authors declare that the research was conducted in the absence of any commercial or financial relationships that could be construed as a potential conflict of interest.
