# Supplementary material for: Sensory Disturbances, but Not Motor Disturbances, Induced by Sensorimotor Conflicts Are Increased in the Presence of Acute Pain
Source: Front Integr Neurosci. 2017 Jul 21;11:14. doi: 10.3389/fnint.2017.00014 (PMC5519624; doi:10.3389/fnint.2017.00014)
Supplement: Supplementary file 1 [file DataSheet1.docx]

Supplementary Material

**Sensory Disturbances, but not Motor Disturbances, Induced by Sensorimotor Conflicts are Increased in the Presence of Acute Pain**

**Clémentine Brun^1,2^, Martin Gagné^1^, Candida S. McCabe^3,4^ and Catherine Mercier^1,2*^**

***Correspondence:**

Catherine Mercier

[Catherine.Mercier@rea.ulaval.ca](mailto:Catherine.Mercier@rea.ulaval.ca)

1. Supplementary Data

Movie

2. Supplementary Figure


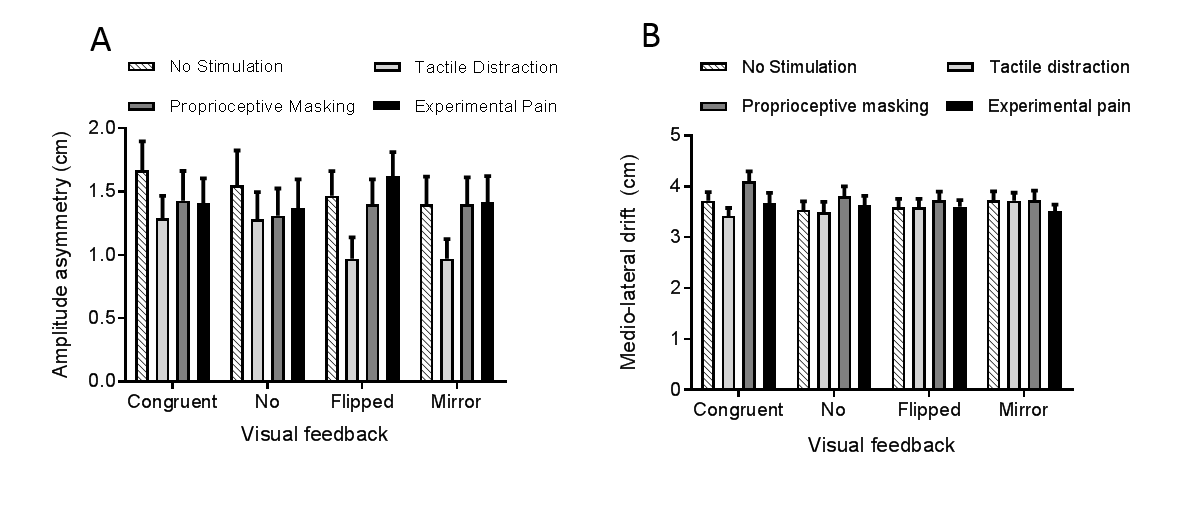


**Figure 1: Amplitude asymmetry (A) and medio-lateral drift (B) during the Baseline phase.** There was no statistical difference between all experimental conditions during the Baseline phase for both motor outcomes.


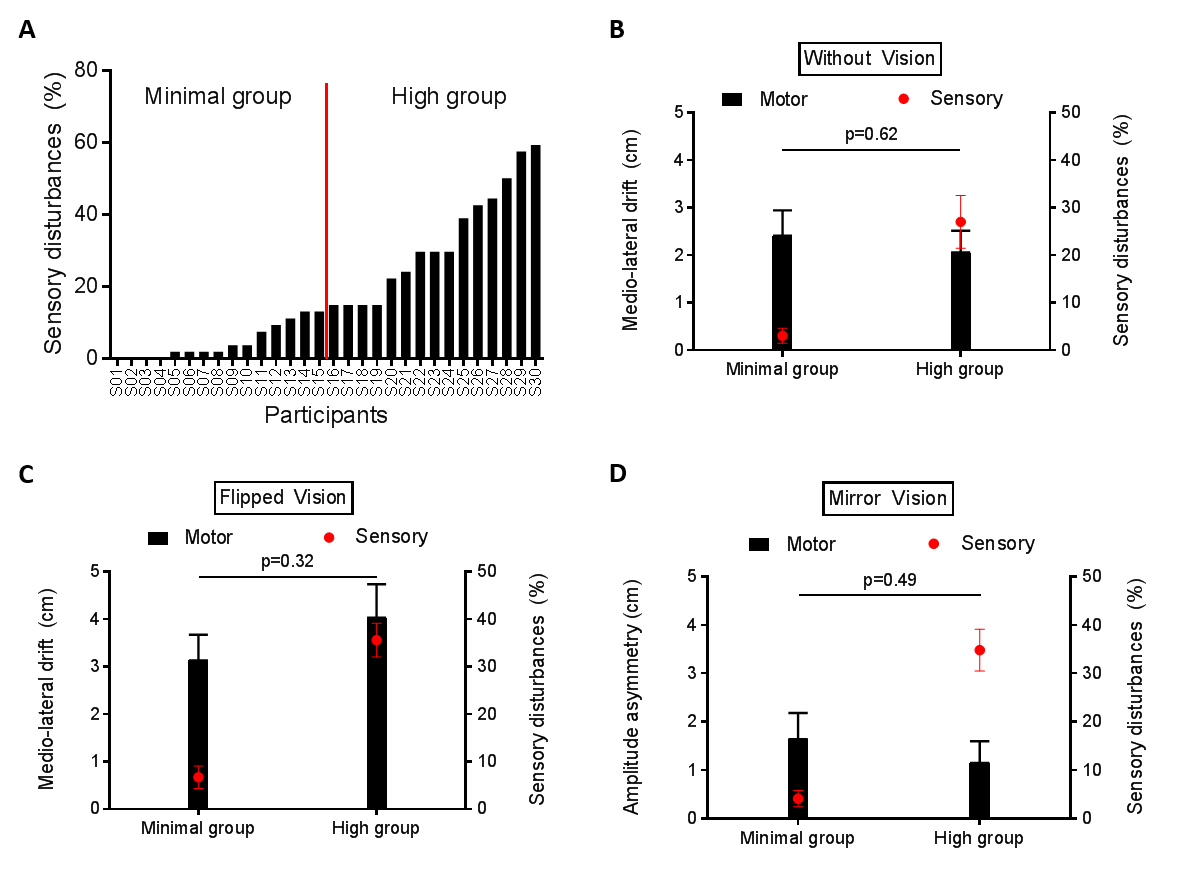


**Supplementary Figure 2**: Motor and sensory disturbances induced by sensorimotor conflict in the Experimental Pain condition. Panel A represents the amount of sensory disturbances across participants for the three sensorimotor conflict conditions during No Stimulation condition. Panel B and C compares the average medio-lateral drift (black bars) and amount of sensory disturbances (red circles) between groups with Minimal vs. High sensory disturbances, in the No VF and Flipped VF conditions, respectively. Panel D compares the average amplitude asymmetry (black bars) and amount of sensory disturbances (red circles) between groups with Minimal vs. High sensory disturbances in the Mirror VF condition. Error bars represent the standard error of the mean. P-values are reported only for motor disturbances (as groups were formed based on amount of sensory disturbances).
